# Is direct anterior approach associated with a compromise of femoral stem survival after total hip arthroplasty? A regional registry-based analysis

**DOI:** 10.1007/s00590-026-04705-1

**Published:** 2026-03-09

**Authors:** Stefanini Niccolò, Morandi Guaitoli Manuele, Bordini Barbara, Mora Paolo, Amabile Marilina, Di Martino Alberto, Faldini Cesare

**Affiliations:** 1 1st Department of Orthopedics and Traumatology, IRCCS Istituto Ortopedico Rizzoli, Bologna, Italy; 2 Medical Technology Lab, IRCCS Istituto Ortopedico Rizzoli, Bologna, Italy; 3 Department of Biomedical and Neuromotor Sciences - DIBINEM - University of Bologna, Bologna, Italy

**Keywords:** Hip Arthroplasty, Approach, Anterior, Stem, Survivorship

## Abstract

**Introduction:**

The direct anterior approach (DAA) for uncemented total hip arthroplasty (THA) has been advocated for early functional recovery, yet concerns persist regarding its influence on femoral component survival. We analysed a large regional registry to compare implant survivorship and the risk of aseptic femoral loosening between DAA and conventional direct lateral/posterolateral (DL-PL) approaches.

**Materials and methods:**

We conducted a retrospective cohort study of 30,753 primary uncemented THAs recorded in the Emilia-Romagna Prosthetic Implant Registry (RIPO) from January 2009 through December 2021. Procedures were classified by surgical approach as DAA (n = 7,663) or DL-PL (n = 23,090). Demographic variables, implant characteristics (including stem length), and reasons for revision were extracted. Kaplan–Meier analysis estimated cumulative survivorship for any-cause revision and for revision due to aseptic loosening; group differences were assessed with the log-rank test. Multivariable Cox proportional hazards models, stratified by age (< 65 vs ≥ 65 years) and adjusted for sex and stem length, evaluated the independent effect of surgical approach on aseptic revision risk. Median follow-up was 4.6 years (IQR 2.3–7.65).

**Results:**

Patients undergoing DAA were slightly younger (mean 67.6 ± 10.4 vs 69.6 ± 9.8 years; *p* 0.001), less frequently obese (21.5% vs 28.7%; *p* < 0.001), and more often received short femoral stems (58.6% vs 24.8%; *p* < 0.001). DAA utilization increased markedly over the study period. Twelve-year cumulative survivorship for all-cause revision was high and similar between groups (DAA 95.6%, 95% CI 94.5–96.7; DL-PL 94.9%, 95% CI 94.4–95.5; log-rank *p* = 0.554). For aseptic loosening, 12-year survivorship was also comparable (DAA 98.9% vs DL-PL 98.4%; *p* = 0.562). In age-stratified adjusted Cox models, surgical approach was not an independent predictor of aseptic revision (age < 65: HR 0.81, 95% CI 0.46–1.44; age ≥ 65: HR 1.32, 95% CI 0.77–2.26).

**Conclusions:**

In this large registry-based series, the DAA was not associated with inferior long-term implant survivorship or a higher risk of aseptic femoral loosening compared with DL-PL approaches after adjustment for patient and implant factors. These findings indicate that, at a population level and with appropriate case and implant selection, DAA yields mechanical outcomes comparable to traditional approaches.

## Introduction

The direct anterior approach (DAA) is becoming increasingly common in total hip arthroplasty (THA), largely due to reports of reduced surgical trauma, reduced pain and bleeding, shorter recovery periods, faster rehabilitation, and improved aesthetic outcomes for patients [1–4].

Despite the increased enthusiasm, DAA performance raised several concerns, particularly related to the technical complexity and the peculiar complications associated with its performance. It is known that the incidence of complications is related to a steep learning curve, which relates with the risk of intra- and postoperative periprosthetic fractures [5–9]. However, recently a potentially higher rate of revisions due to stem malpositioning in DAA patients has been reported [10, 11]. A recent meta-analysis comparing DAA with the direct lateral (DL) approach confirmed this trend, showing that elevated rates of dislocation, prosthetic loosening, and periprosthetic fractures were observed in the DAA group [12]. Further studies have shown an elevated incidence of femoral stem loosening and radiographic evidence of suboptimal fixation in DAA, contributing to an increased risk of early aseptic loosening and requirement of revision surgery [13–15].

These complications have been largely attributed to the technical challenges specific to femoral exposure in DAA [16], which may hinder accurate implant positioning and increase the likelihood of varus alignment or undersized stem placement, particularly during the surgeon’s learning curve [17–19].

In view of the growing popularity of DAA and the concerns that this raises regarding early mechanical failure, we designed a retrospective registry-based study inquiring the survival of THA implants based on the used surgical approach, mainly regarding the occurrence if failure because of aseptic loosening.

## Materials and methods

This retrospective cohort study drew upon the Emilia-Romagna Regional Registry of prosthetic implants (RIPO) to evaluate 30,753 primary uncemented total hip arthroplasties (THAs) implanted between January 2009 and December 2021. RIPO captures more than 98% of all regional arthroplasty procedures, provided detailed patient, implant and surgical-approach data under compulsory reporting by both public healthcare structures and private institutions.

### Inclusion and exclusion criteria

We included all patients resident in Emilia-Romagna who underwent primary uncemented THA for primary hip osteoarthritis via either the direct anterior approach (DAA) vs other “classical” approaches (direct lateral or posterolateral) performed between January 2009 and December 2021. All THAs implanted before 2009 were excluded from the analysis, as 2009 marks the first year in which a substantial number of procedures performed using DAA were documented.

Other exclusion criteria included any procedure involving dual-mobility cups, modular femoral stems, or large-head metal-on-metal bearings (≥ 36 mm). Stem design (short vs. long) and bearing surfaces were recorded, when available.

### Patient demographics and characteristics of the implants

Data extraction, completed in April 2025, yielded a cohort consisting of 7,663 cases in the DAA group and 23,090 classical approaches. Detailed patient demographics—including age distribution, sex and body mass index (BMI) surgical approach and implant characteristics—were recorded.

### Data analysis

Patient demographics, implant features, and the reasons for revision (the primary endpoint) were analysed using descriptive statistics. Continuous variables were summarized as mean ± SD and median with interquartile range, categorical variables as counts and percentages. Implant survival—defined both as revision for any cause and as revision for aseptic stem or total component loosening—was estimated by Kaplan–Meier analysis with censoring at death or 31 December 2021. Differences between survival curves were assessed using the Log-rank test. Threshold for significance was *P* = 0.05. To adjust for potential confounders, a multivariate Cox proportional-hazards model stratified by age (< 65 years vs. ≥ 65 years) was fitted, incorporating sex, surgical approach and stem length as covariates. The Wald test was conducted to analyze the *p*-values for data achieved from the Cox multiple regression analyses. The proportional hazards assumption was estimated using the Schoenfeld residual method. The proportional hazards assumption was estimated using the Schoenfeld residual method. Statistical analyses were conducted using R Core Team (2024). R: A Language and Environment for Statistical Computing_. R Foundation for Statistical Computing, Vienna, Austria https://www.R-project.org/, and JMP, version 12.0.1 (SAS Institute Inc., Cary, NC, USA, 1989–2007).

### Ethical consideration

As this study used pseudoanonomized registry data collected during routine practice; therefore, formal ethical committee approval was unnecessary.

## Results

The cohort included 7663 patients in the DAA group (median age 69.0 years, IQR 61.0–75.0; 54.1% female) and 23,090 in the classical-approach group (median age 71.0 years, IQR 64.0–77.0; 52.3% female). Patients undergoing DAA were slightly but significantly younger (mean ± SD 67.6 ± 10.4 years vs. 69.6 ± 9.8 years; *p* < 0.001) and had lower prevalence of obesity (21.5% vs. 28.7%; *p* < 0.001) (Table 1).

**Table 1:** Demographic characteristics of patients included in the study, including age, gender, and BMI

	DAA N = 7,663^1^	DL-PL N = 23,090^1^	*p*-value^2^
Age			< 0.001
Median (Q1, Q3)	69.0 (61.0, 75.0)	71.0 (64.0, 77.0)	
(Min, Max)	(26.0, 95.0)	(16.0, 96.0)	
Mean (SD)	67.6 (10.4)	69.6 (9.8)	
Age class			< 0.001
< 40	46 (0.6)	106 (0.5)	
40–49	398 (5.2)	768 (3.3)	
50–59	1,235 (16.1)	2,681 (11.6)	
60–69	2,384 (31.1)	6,739 (29.2)	
70–79	2,665 (34.8)	9,448 (40.9)	
80 +	935 (12.2)	3,348 (14.5)	
Age groups			< 0.001
< 65	2,737 (35.7)	6,334 (27.4)	
≥ 65	4,926 (64.3)	16,756 (72.6)	
Gender			0.007
Female	4,145 (54.1)	12,079 (52.3)	
Male	3,518 (45.9)	11,011 (47.7)	
BMI			< 0.001
Underweight	58 (0.9)	126 (0.6)	
Normal Weight	1,972 (30.1)	5,088 (25.4)	
Overweight	3,108 (47.5)	9,091 (45.3)	
Obese	1,408 (21.5)	5,762 (28.7)	
Unknown	1,117	3,023	

^1^ n (%)

^2^ Welch Two Sample t-test; Pearson’s Chi-squared test

The distribution of femoral-head sizes and bearing materials also differed significantly between groups, as did the proportion of short vs. long stems (58.6% short in DAA vs. 24.8% short in classical; *p* < 0.001) (Table 2).

**Table 2:** Characteristics of prosthetic implants in the study groups in terms of head dimension, bearing material and coupling (CoC, ceramic on ceramic; CoP, ceramic on polyethylene; MoP, metal on polyethylene; MoM, metal on metal), and size of the stems

	DAA N = 7,663^1^	DL-PL N = 23,090^1^	*p*-value^2^
Head diam			< 0.001
< 36 mm	3,070 (40.1)	9,908 (42.9)	
≥ 36 mm	4,593 (59.9)	13,175 (57.1)	
Unknown	0	7	
Art. coupling			< 0.001
CoC	5,162 (67.5)	10,202 (44.3)	
CoP	2,409 (31.5)	10,329 (44.8)	
MoP	67 (0.9)	1,984 (8.6)	
MoM	1 (0.0)	315 (1.4)	
Other	9 (0.1)	216 (0.9)	
Unknown	15	44	
Stem lenght			< 0.001
Long	3,176 (41.4)	17,365 (75.2)	
Short	4,487 (58.6)	5,725 (24.8)	

^1^ n (%)

^2^ Welch Two Sample t-test; Pearson’s Chi-squared test

**Fig. 1:**
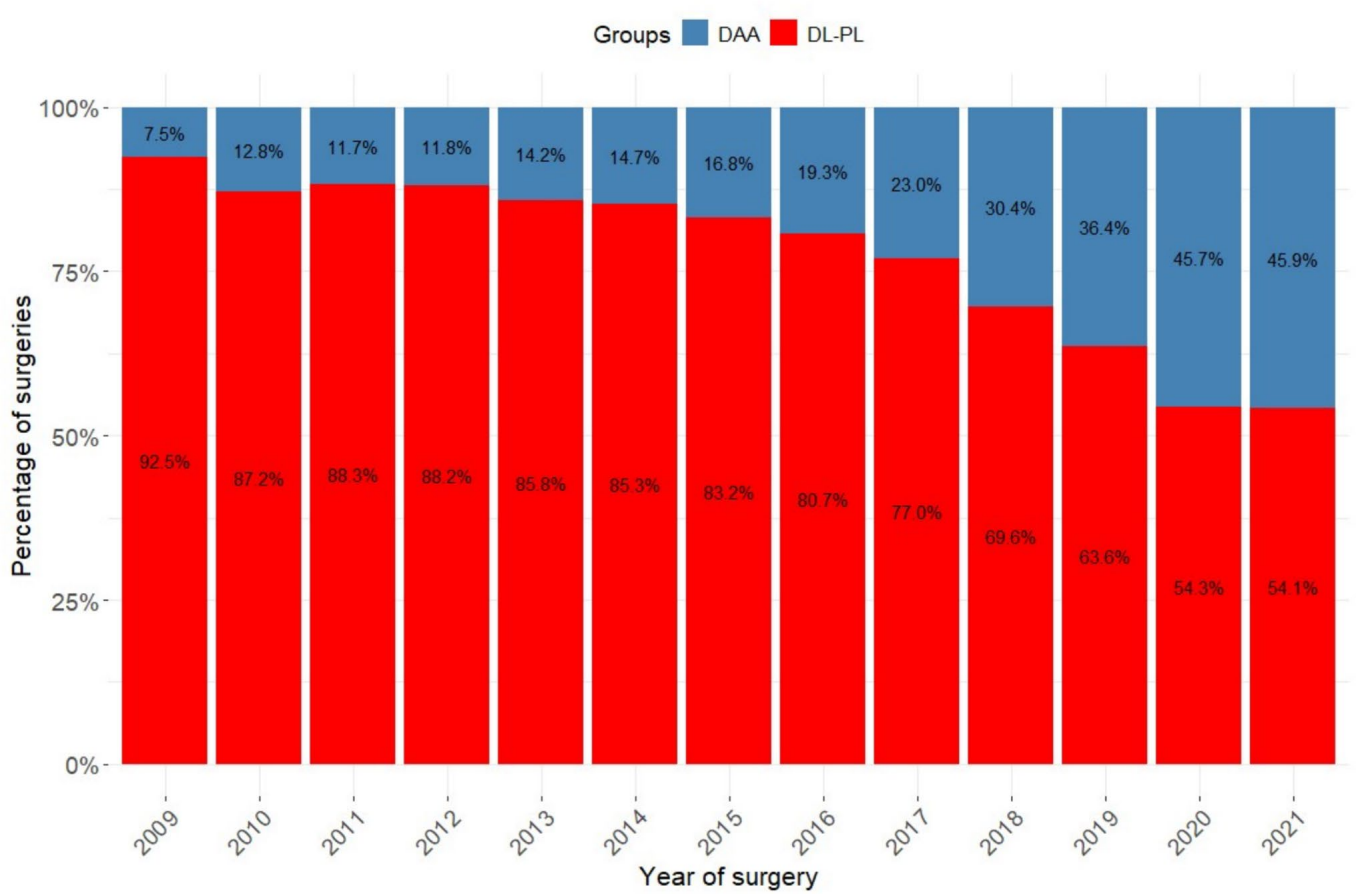
Bar chart depicting the annual utilization trends of the various surgical approaches under examination, demonstrating a steady increase in the use of the DAA compared with the other classical approaches

Over the 13-year study period, the adoption of the DAA increased progressively, and it accounted for 7.5% of uncemented THAs in 2009, reaching 45.9% of all the primary THA implants by 2021, while the use of classical approaches (DL-PL) declined in parallel (Fig. 1).

**Fig. 2:**
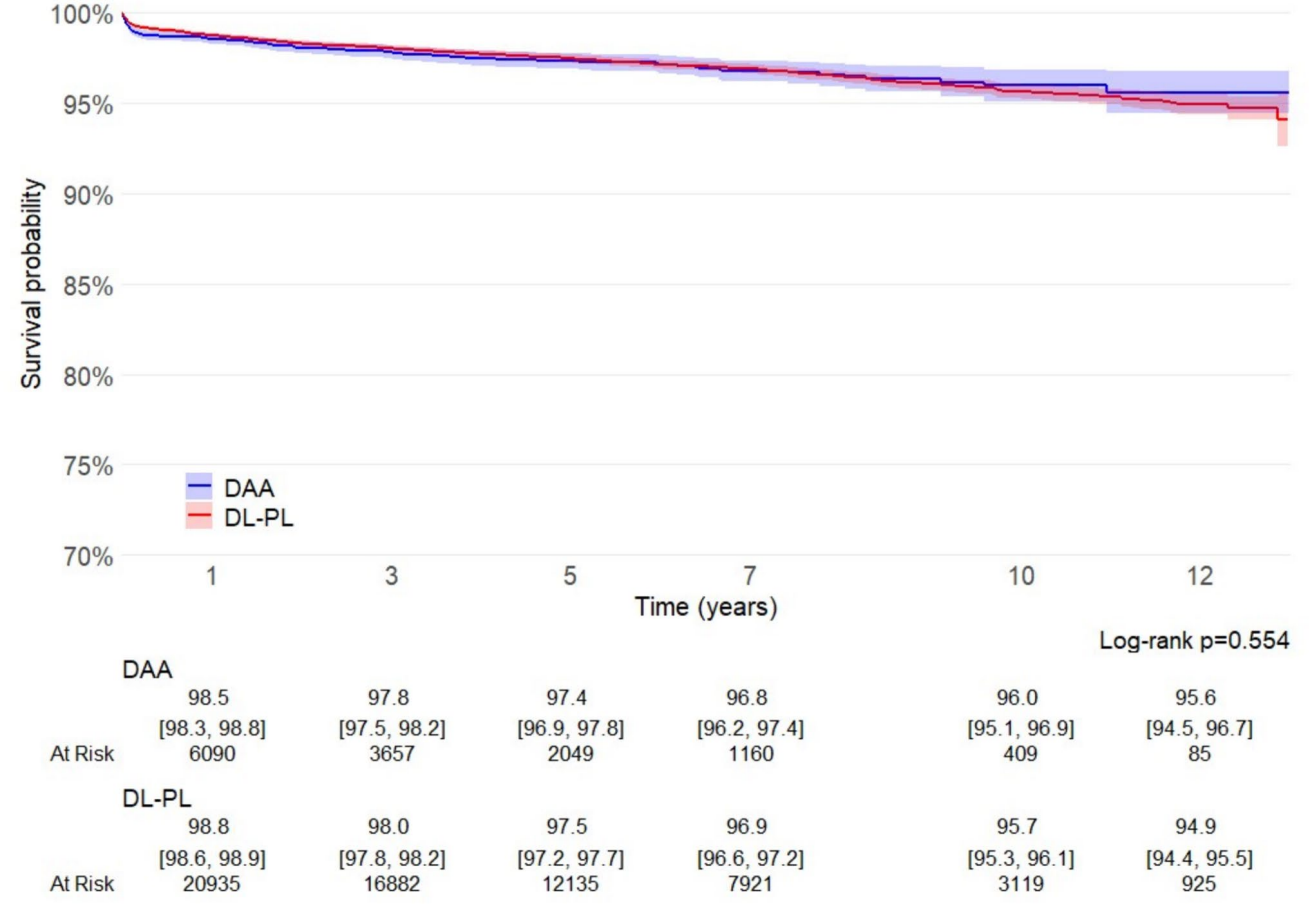
Survival curves for all causes of failure, calculated and plotted according to the Kaplan–Meier method

Median follow-up for the entire cohort was 4.6 years (IQR 2.3–7.65). Kaplan–Meier analysis revealed no significant difference in the overall implant survival between DAA and DL-PL (log-rank *p* = 0.554), with 12-year cumulative survivorship exceeding 94% in both groups (DAA 95.6%, 95% CI 94.5–96.7; DL-PL 94.9 CI 94.4–95.5; p = 0.554) (Fig. 2).

**Fig. 3:**
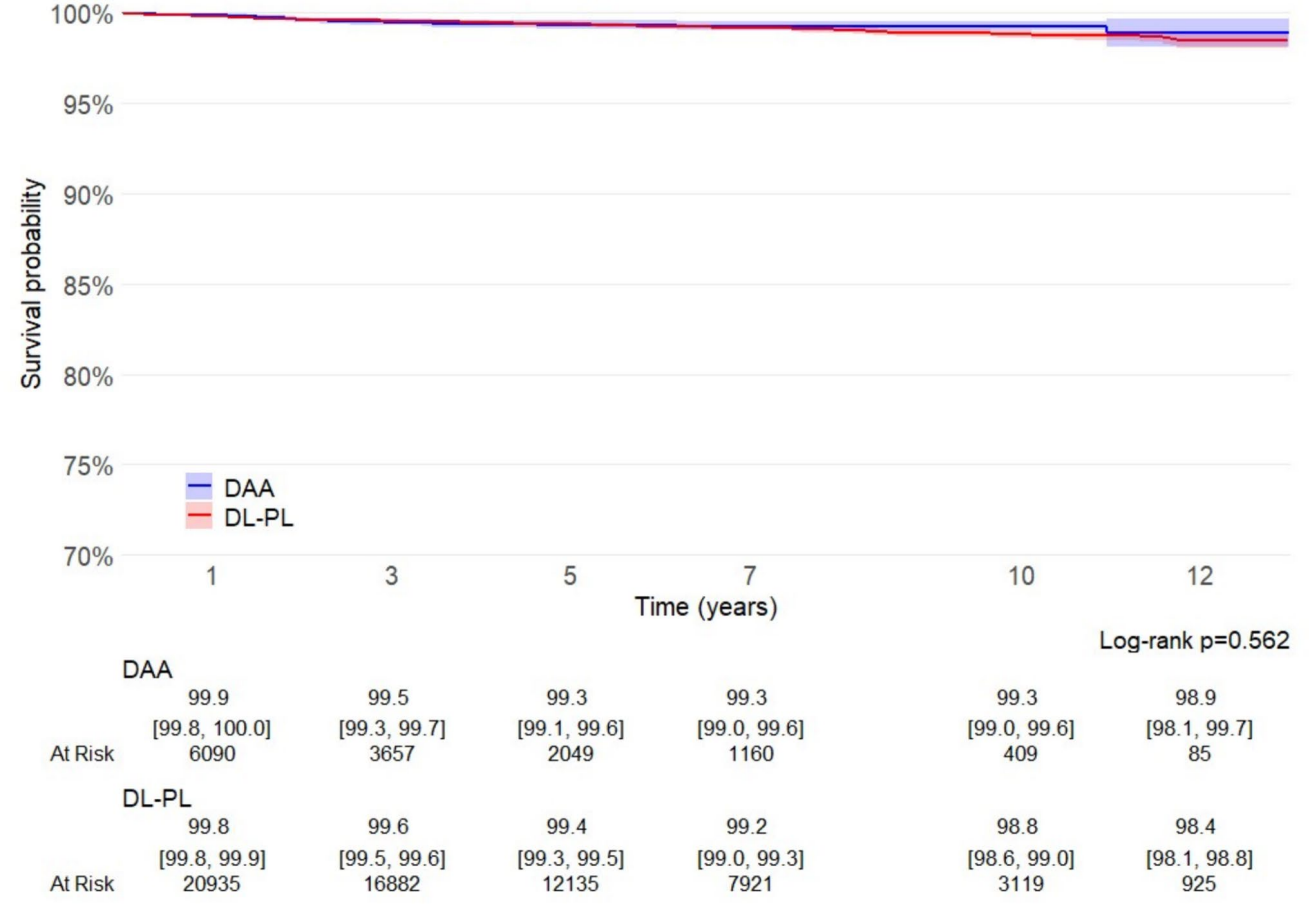
Survival curves for failures due to aseptic loosening of the stem or total component, calculated and plotted according to the Kaplan–Meier method

When the endpoint was restricted to aseptic loosening of the stem or combined cup and stem loosening, twelve-year survival remained elevated, with no significant differences among compared groups (DAA 98.9%, 95% CI 98.1–99.7; DL-PL 98.4%, 98.1–98.8; *p* = 0.562) (Fig. 3).

In multivariate Cox proportional-hazards models adjusting for sex and stem length and stratified by age (< 65 vs ≥ 65 years), surgical approach did not emerge as a predictor of aseptic revision. For patients younger than 65years, the HR for classical versus DA approach was 0.81 (95% CI 0.46–1.44; p = 0.474), whereas in those aged 65 or older it was 1.32 (95% CI 0.77–2.26; *p* = 0.306), confirming no significant difference in long-term mechanical performance between access routes (Table 3).

**Table 3:** Cox regression model for aseptic loosening of the stem or total component, adjusted for age, sex, incision type, and stem length

Age < 65 years	HR^1^	95% CI^1^	p-value	Age ≥ 65 years	HR^1^	95% CI^1^	*p*-value
Gender				Gender			
Female	–	–		Female	–	–	
Male	1.33	0.83, 2.12	0.237	Male	1.55	1.09, 2.21	0.015
Approach				Approach			
DAA	–	–		ANT	–	–	
DL-PL	0.81	0.46, 1.44	0.474	DL-PL	1.32	0.77, 2.26	0.306
Stem lenght				Stem lenght			
Long	–	–		Long	–	–	
Short	0.65	0.39, 1.10	0.106	Short	1.01	0.67, 1.52	0.970
^1^HR = Hazard ratio, CI = Confidence interval				^1^HR = Hazard ratio, CI = Confidence interval			

## Discussion

In the current study, on a large cohort of uncemented THAs implants, no difference was observed in implant stem survival at long-term, when DAA and traditional DL-PL approaches were compared. In this large cohort, patients undergoing DAA were slightly younger and less frequently obese than those treated via classical approaches. This tendency probably reflects a strategy for approaching DAA, particularly during the initial phase of the learning curve, through more careful case selection, as recommended in literature [3, 20]. Notably, DAA increased from 8% in 2009 to 48% in 2021, at the expenses of DL and PL approaches. Our Kaplan–Meier analysis did not show significant difference neither in 10-year overall implant survival between DAA (96.0%, 95% CI 95.1–96.9) and DL-PL (95.7%, 95% CI 95.3–96.1; log-rank p = 0.554) (Fig. 2), nor for aseptic loosening (DAA 99.3% vs. DL-PL 98.8%, *p* = 0.562) (Fig. 3).

These findings contrast with earlier reports of increased early stem failures and aseptic loosening associated with DAA in smaller series. Eto et al. [13] analysed early revision surgeries in 130 primary THAs and reported a significantly higher incidence of femoral stem loosening in procedures performed via DAA compared to those using non-anterior approaches. Similarly Angerame et al. [14] found that although overall revision rates were not significantly different among approaches, but DAA was associated with a greater incidence of femoral loosening compared to the posterior approach (PA). Conversely, Haugan et al. [21] reported no significant differences in terms of stem subsidence among DL, DAA, and PA.

Meneghini et al. [22] in a multicenter retrospective review of 342 cases of early femoral failure after cementless THA, reported that 50.9% of these failures occurred following the direct anterior approach (DAA), versus 34.8% after the direct lateral approach (DL) and 14.3% after the posterior approach (PA) (*p* = 0.001), and demonstrated through multivariate analysis that DAA is an independent predictor of early femoral failure.

Considering a wider population, an analysis of 122,345 primary THAs from the Australian Orthopaedic Association National Joint Replacement Registry demonstrated that the DAA is associated with a significantly increased risk of femoral complications, particularly revisions due to periprosthetic femoral fractures and aseptic loosening of the femoral stem, compared to both posterior and lateral approaches [23]. In a subsequent study involving 60,739 cemented femoral stems, the anterior approach did not show a higher incidence of revision for periprosthetic fracture; however, it was associated with a substantially increased risk of femoral stem loosening, with a hazard ratio of 5.06 (95% CI: 3.08–8.30; *p* < 0.001) when compared to the posterior approach [24].

Our findings are in contrast with previously reported literature: multivariate Cox models—adjusted for sex, stem length, and stratified by age (< 65 vs. ≥ 65 years)—revealed no significant hazard associated with either approach for aseptic revision (HR 0.81 in < 65 years, *p* = 0.474; HR 1.32 in ≥ 65 years, *p* = 0.306). Accordingly to our findings, Mjaaland et al. [25] analyzing data from the Norwegian Arthroplasty Register involving 21,860 uncemented THAs, found no significant differences in 5-year survival rates or revision risks across different surgical approaches.

Garavaglia et al. [15] documented radiographic signs of suboptimal stem fixation after DAA, correlating with early aseptic revision. Our registry-based design precludes systematic radiographic evaluation, yet the equivalence in long-term survival suggests that any possible subtle fixation deficits are, on average, clinically inconsequential.

Technical challenges inherent to femoral exposure in DAA—such as insufficient capsular release leading to varus and anteverted alignment and stem undersizing—have been proposed as mechanisms for early failures. Haversath et al. [17] reported higher varus alignment rates with straight tapered stems during the DAA learning phase, Sidhu et al. [11] reported a tendency of greater anteversion of femoral stem through DAA compared to PL, while Rivera et al. [18] described a greater tendency to undersize stems versus PA. Interestingly Haversath et al. [26] demonstrated that a lower critical trochanter angle (mean 25.0° ± 7.5°) correlated negatively with stem alignment (r = –0.52; p ≤ 0.001), independent of approach, suggesting that a varus stem positioning is driven more by patient-specific proximal femoral anatomy than by the choice of surgical approach.

Macheras et al. [27] attributed a cluster of aseptic loosening to a single femoral stem design (Quadra-S™, Medacta International, Strada Regina—6874 Castel San Pietro—Switzerland), implicating that implant surface characteristics—rather than surgical approach— is involved in early failures. The influence of stem design on early and mid-term stability extends beyond isolated series. Sershon et al. [28] reported that collared and fit-and-fill stem geometries reduced periprosthetic fracture risk within 90 days, regardless of approach. The foregoing observations are congruent with those of Rele et al. [29], who showed that collarless cementless stems in DAA carry substantially higher revision risks than collared designs—nearly double for all‐cause revision at three months (HR 1.99), almost triple the risk of revision for periprosthetic fracture in six months (HR 2.90), over a ten-fold risk after six months (HR 10.04), and a markedly higher risk of aseptic loosening–related revision at two years (HR 5.76). Wagner et al. [30] affirm that modern, shorter double-taper stems with minimized lateral shoulders facilitate implantation via the muscle-sparing DAA, yet some designs still exhibited higher revision rates, predominantly for periprosthetic fracture (1.5%) and aseptic loosening (1.4%). Cidambi et al. [31] in a retrospective review of 1,120 consecutive DAA THAs, found that collared, fully hydroxyapatite-coated, and triple-tapered stems provided superior stability and lower aseptic loosening risk compared with short, collarless, mediolaterally tapered designs. A study evaluated stem survival across Mont classification types in 53,626 uncemented THAs in the same regional register of RIPO [32]. Modest differences were reported—failure rates ranged from 1.6% (type 1) to 3.9% (type 6)—mainly caused by aseptic loosening, which was the most common complication in type 2 (double wedge) stems and periprosthetic fractures, and the most common complication in type 6 (anatomic stems). This underscores that implant choice—particularly stem design and surface coating—may exert a greater influence on uncemented THR outcomes than the choice of surgical approach.

These findings further support our results, indicating that the DAA yields long-term outcomes comparable to traditional DL-PL approaches. Moreover, implant selection, particularly femoral stem design, seems to play a more relevant role in determining the success of uncemented THA than the surgical approach itself.

## Limitations

Despite the strengths of large sample size, extended follow-up, and rigorous statistical adjustment, this study has limitations, typically connected to the intrinsic nature of a registry study. The observational design cannot eliminate residual confounding factors including surgeon experience, patient selection, or unmeasured variables such as bone quality and perioperative rehabilitation. Implant selection and surgical technique likely evolved over the study period, potentially biasing early versus late outcomes. Finally, functional and patient-reported outcome measures were not available, precluding assessment of clinical recovery, soft-tissue preservation, or complications such as lateral thigh pain and dislocation rates.

## Conclusion

In summary, despite the inherent limitations of registry-based observational studies, our analysis of a large cohort of uncemented total hip arthroplasties demonstrates no significant difference in long-term implant survival between the DAA and traditional DL-PL techniques. Rather, implant-related factors—most notably femoral stem design and surface characteristics—emerge as stronger determinants of outcome. The observed equivalence in implant survival reinforces the notion that with appropriate patient selection, surgical expertise, and optimized implant choice, DAA can yield outcomes comparable to those of more traditional approaches. Accordingly, concerns regarding DAA-related early failures should be contextualized within the broader framework of surgical learning curves, anatomical variability, and stem-specific performance.

## Data Availability

No datasets were generated or analysed during the current study.
